# Elevated Circulating hsa-miR-106b, hsa-miR-26a, and hsa-miR-29b in Type 2 Diabetes Mellitus with Diarrhea-Predominant Irritable Bowel Syndrome

**DOI:** 10.1155/2016/9256209

**Published:** 2016-08-22

**Authors:** Wenhua Tao, Xiaoyun Dong, Guimei Kong, Penghua Fang, Xiaoli Huang, Ping Bo

**Affiliations:** Jiangsu Key Laboratory of Integrated Traditional Chinese and Western Medicine for Prevention and Treatment of Senile Diseases, Medical School of Yangzhou University, No. 11 Huaihai Road, Yangzhou, Jiangsu 225001, China

## Abstract

*Background and Aims*. Although the differential expression of microRNA (miRNA) genes has been identified in many diseases, little information exists concerning the miRNA expression profile in type 2 diabetes mellitus (T2DM) with diarrhea-predominant irritable bowel syndrome (D-IBS). Therefore, the specific expression of miRNAs in diabetes with D-IBS is identified in the study.* Materials and Methods*. 201 patients with IBS and 220 matched healthy controls were included in the study. Microarray technology and real-time reverse transcriptase-polymerase chain reaction analysis (RT-PCR) were taken to examine the miRNA expression profiles of T2DM patients with diarrhea-predominant irritable bowel syndrome (D-IBS) compared with patients with T2DM, patients with D-IBS, and control subjects.* Results*. We have found that 35 miRNAs were differentially expressed in T2DM with D-IBS, in which three representative miRNAs, hsa-miR-106b, hsa-miR-26a, and hsa-miR-29b, were found to be significantly elevated in T2DM with D-IBS by RT-PCR.* Conclusions*. Our study has indicated that hsa-miR-106b, hsa-miR-26a, and hsa-miR-29b were elevated in T2DM with D-IBS, which may be the potential biomarkers of T2DM with D-IBS. To obtain a better understanding of the biological functions of these miRNAs in T2DM with D-IBS, functional annotation analysis suggested that the MAPK pathway may be responsible for T2DM with D-IBS.

## 1. Introduction

Irritable bowel syndrome (IBS) is a common gastrointestinal (GI) disorder that is characterized by chronic abdominal pain and changes in bowel habits, including the frequent occurrence of diarrhea (D-IBS), constipation (C-IBS), or a combination of both (M-IBS) [1, 2]. Recent studies have reported that approximately 10–20% of adults in Western countries have IBS symptoms, and similar prevalence has been reported in Asia [3–5]. IBS is not a fatal disease, but it greatly reduces quality of life [6, 7].

Diabetes mellitus (DM) is a group of metabolic diseases which are characterized by hyperglycemia, resulting from defects in insulin secretion, insulin action, or both [8]. The chronic hyperglycemia of diabetes is associated with long-term damage, dysfunction, and failure of various organs [9]. Previous studies indicate that approximately 70–75% of diabetic patients have a least one gastrointestinal symptom [10, 11]. Besides, the frequency of prediabetes in patients with irritable bowel syndrome was higher than that in the matched controls [12]. Glucose control and IBS are closely linked to each other. On one hand, hyperglycemia is known to impair gastric and small intestinal motility, possibly through vagal-cholinergic neural inhibition or by altering serum osmolality and gastrointestinal peptide secretion [13]. On the other hand, gastrointestinal motility disorders, such as IBS, may give rise to postprandial glycemic dysregulation.

miRNAs are endogenously expressed, evolutionarily conserved, small single-stranded noncoding RNAs of approximately 22 nucleotides in length which fine-tune gene expression [14] and have piqued interest as diagnostic biomarkers, indicators of etiology, and potential therapeutic targets [15]. Since their discovery in the nematode* Caenorhabditis elegans* in 1993 [16], thousands of miRNAs have been identified [17]. miR-29 is routinely examined in the study of type 2 diabetes mellitus (T2DM) [18, 19] and IBS [20, 21]. MicroRNA-26a regulates insulin sensitivity and the metabolism of glucose and lipids [22], and microRNA-106b is reported to correlate closely with skeletal muscle insulin resistance and T2DM [23], but there are little studies of T2DM with D-IBS that have focused on miRNA expression.

Most studies of gene expression in IBS and T2DM focus on intestinal gene expression; these require invasive sampling of the biopsies. The purpose of this preliminary investigation was to examine circulating miRNA expression as a less invasive measure of molecular dysregulation and conduct the correlative analysis in T2DM with D-IBS.

## 2. Materials and Methods 

### 2.1. Study Subjects

The subjects of the study were two hundred and one patients with IBS (age: 18–75 years) who were diagnosed according to Rome III criteria without organic disease [24]. During the introductory session, participants underwent (1) physical examination, (2) lactulose breath test for bacterial overgrowth, and (3) blood draw for tissue transglutaminase antibody to rule out coeliac sprue, and those who had IBS symptoms for at least 1 year were recruited for this study. The control group included 220 healthy individuals who were matched to cases based on age, sex, and body mass index (BMI). We excluded all cases and controls with T2DM that were based on the results of the oral glucose tolerance test. The main characteristics of all participants are described in Table 1. The study was approved by the Ethics Committee of Affiliated Hospital to Yangzhou University (Jiangsu, China), and written informed consent was obtained from each subject.

### 2.2. Sample Collection, Isolation of Plasma, and RNA Extraction

Venous blood samples (5 mL) from all participants were collected by standard venipuncture in Kangjian® tubes containing EDTA and immediately centrifuged at 3000 ×g for 30 min at room temperature, and the supernatants were then centrifuged at 13000 ×g for 5 min at 4°C. The supernatants from each subject were stored at −80°C until they were prepared for analysis for cell-free miRNA expression. Total RNA was extracted from the plasma using a mirVana*™* RNA isolation Kit (Ambion, Austin, TEXAS, USA) in accordance with the manufacturer's protocol. The purity and the concentration of RNA samples were determined using a NanoDrop ND-2000 Spectrophotometer (NanoDrop Products, Wilmington, DE, USA), and their integrity was assessed by an Agilent Bioanalyzer 2100 (Agilent Technologies, Santa Clara, CA). RNA samples that were free of protein and phenol and presented an RNA integrity number ≥ 7.0 were considered for microarray analysis.

### 2.3. MicroRNA Microarray Analysis

Three patients were selected randomly from each group for microRNA microarray analysis. Plasma microRNA profiling was performed using a Human miRNA Microarray kit, 8 × 60K (based on Sanger miRbase release 19.0, Design ID: 046064, Agilent Technologies, Santa Clara, CA). The miRNA Complete Labeling and Hyb. Kit (Agilent Technologies, Santa Clara, CA) was used for labeling and hybridization of 100 ng of total RNA, according to the manufacturer's instructions. Briefly, total RNAs were dephosphorylated using calf intestinal phosphatase, denatured using dimethylsulfoxide (DMSO), and then labeled with Cyanine 3-CTP using T4 RNA ligase for 2 h at 16°C and then at 55°C in a hybridization oven for 20 min. After purification, the labeled RNAs were hybridized onto the microarray. After washing, the arrays were scanned with an Agilent Scanner G2505C (Agilent Technologies, Santa Clara, CA).

### 2.4. Quantitative Real-Time PCR

The levels of miRNAs were detected by real-time reverse transcriptase-polymerase chain reaction analysis. The cDNA was generated using a FastQuant RT kit (with gDNase) (TIANGEN Biotech, Beijing, China). The transcribed cDNA was diluted 50 times with DNase-free water, and real-time quantitative RT-PCR (qRT-PCR) was performed using a 7500 Real-Time PCR System (Ambion, Austin, Texas). The determined threshold cycle (CT) was normalized with U6 as an endogenous control, and the relative amounts of miRNAs expression in different groups were determined using a comparative CT method. The primers used were listed in Table 2.

### 2.5. Microarray Analysis

Feature Extraction software (version 10.7.1.1, Agilent Technologies) was used to obtain raw data and analyze the array images. GeneSpring software (version 12.5, Agilent Technologies) was employed to complete the basic analysis with the raw data. And the raw data were normalized using the quantile-filling algorithm. The probes with at least 100.0 percent of samples in any 1 of 2 conditions were flagged as “detected” and were chosen for further data analysis. Differentially expressed miRNAs were identified by fold changes as well as by *p* values that were calculated by *t*-test. The threshold that was set for upregulated and downregulated genes was a fold change ≥ 2.0 and a *p* value ≤ 0.05. The target genes of differentially expressed miRNAs were predicted by three databases (TargetScan, microRNA.org, and PITA). GO and KEGG analyses were applied to determine the effect of these target genes. Hierarchical clustering was performed to demonstrate the distinguishable miRNA expression patterns among the samples.

### 2.6. Statistical Analysis

All values are expressed as the mean ± SD. All experiments were repeated at least three times. Statistical differences between two groups were determined using Student's *t*-test. Two-way analysis of variance (ANOVA) with general linear model procedures using a univariate approach was applied for more than two groups. *p* value < 0.05 was considered statistically significant.

## 3. Results 

### 3.1. Demographic and Clinical Data

The 201 patients in the IBS group were composed of 128 (63.7%) females and 73 (36.3%) males, whereas the 220 controls were composed of 128 (58.2%) females and 92 (41.8%) males (Table 1). The IBS group consisted of 134 D-IBS and 67 C-IBS patients. The IBS group was not significantly different in terms of age, sex, and BMI compared to healthy controls. The clinical characteristics of each patient group are listed in Table 1. Fasting blood glucose was significantly higher in IBS patients compared to healthy subjects (Table 1).

### 3.2. Distinct miRNA Expression Signatures in Plasma from T2DM with D-IBS Patients

**Figure 1:**
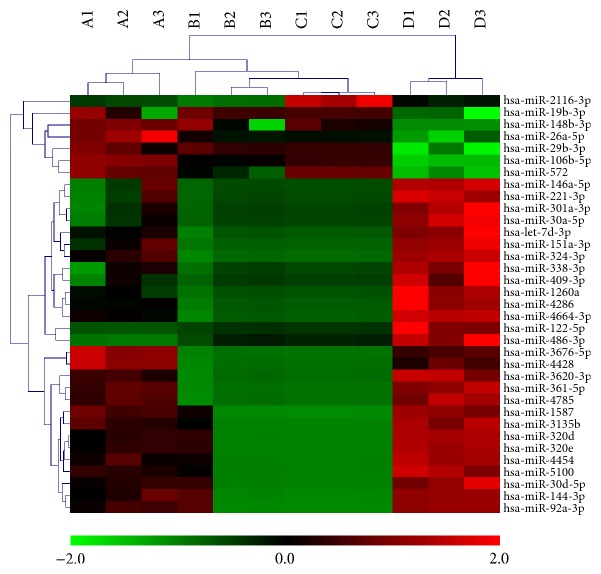
Clustering and principle component analysis of 35 miRNAs with significantly different expression values (*p* value < 0.05; FC ≥ 2). Unsupervised hierarchical clustering analysis of 35 miRNAs with significantly different expression values in T2DM with D-IBS (A), T2DM (B), and D-IBS (C) compared with the matched samples (D). One through three represent three different patients for each group. The color key under the heat map represents the different expression levels. Expression levels of miRNAs are shown in red (upregulated) and green (downregulated), with brighter shades indicating higher fold differences.

We compared the miRNA expression profiles of the plasma from three T2DM patients with D-IBS, three T2DM patients, and three D-IBS patients with those of three matched subjects by performing miRNA microarray experiments. A total of 91 unique probes exhibited significant differences (*p* value ≤ 0.05 and FC ≥ 2.0) in expression levels between the T2DM patients and the control group, and 245 unique probes exhibited significant differences between the IBS-D and the control groups. Thus, compared with the control group, 314 probes exhibited difference when used in T2DM with D-IBS. Among the expressional expression of miRNAs in three groups, only 35 were identified which significantly expressed miRNAs in T2DM with D-IBS compared with D-IBS and T2DM, including hsa-miR-106b, hsa-miR-26a, and hsa-miR-29b, and also passed the area under roc curve (AUC) threshold of 0.90, ranging from 0.90 to 1.00. The values for fold changes and *p* for each of the 35 microRNAs are presented in Table 3. Then, the results were visualized by performing hierarchical clustering analysis (Pearson uncentered distance metric with average linkage) using the normalized expression values of those miRNAs. It can be seen that the miRNAs possess a discriminatory power that can distinguish T2DM patients with D-IBS from patients with D-IBS, patients with T2DM, and healthy individuals, as all patients were clustered together and were separated from the control subjects (Figure 1).

### 3.3. Differential Expression of hsa-miR-106b, hsa-miR-26a, and hsa-miR-29b in T2DM with D-IBS

**Figure 2:**
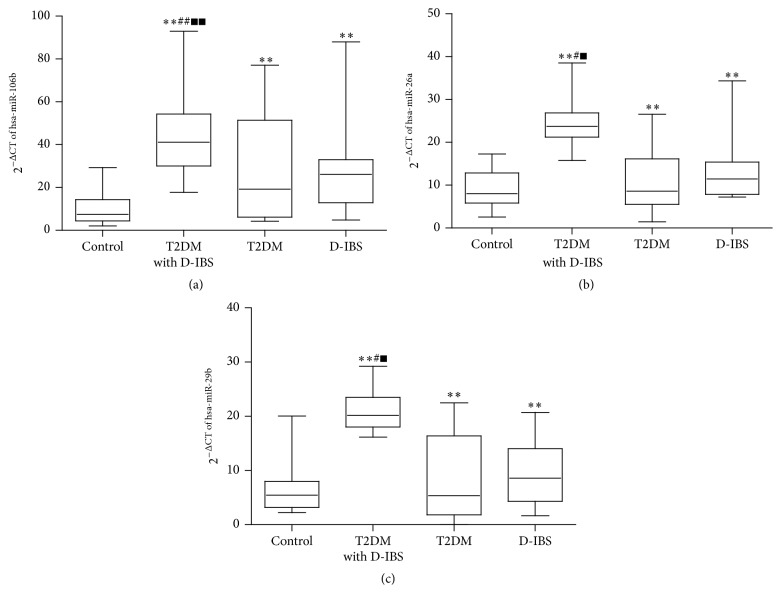
Box plots showing the differential expression of (a) hsa-miR-106b, (b) hsa-miR-26a, and (c) hsa-miR-29b in healthy controls and in patients with T2DM, D-IBS, and T2DM with D-IBS. ^*∗∗*^ *p* < 0.01 versus control; ^##^ *p* < 0.01 and ^#^ *p* < 0.05 versus T2DM; ^■■^ *p* < 0.01 and ^■^ *p* < 0.05 versus D-IBS.

After analysis of the microRNA microarray results, three important miRNAs, hsa-miR-106b, hsa-miR-26a, and hsa-miR-29b, were confirmed by real-time reverse transcriptase-polymerase chain reaction analysis. A total of 72 plasma samples were obtained from T2DM with D-IBS, T2DM, D-IBS, and normal healthy control subjects. We performed quantitative real-time PCR on the total RNAs that were isolated from these plasma samples. We found that the expression of hsa-miR-106b, hsa-miR-26a, and hsa-miR-29b was significantly elevated in T2DM with D-IBS, T2DM, and D-IBS compared to the control subjects (Figures 2(a), 2(b), and 2(c)). However, the levels of hsa-miR-106b, hsa-miR-26a, and hsa-miR-29b expression were significantly different in T2DM with D-IBS compared to either T2DM or D-IBS (*p* < 0.05) (Figure 2).

### 3.4. Prediction of the Targets of the Above-Mentioned Three miRNAs and Biological Significance and Pathway Analyses of the miRNA Targets

**Figure 3:**
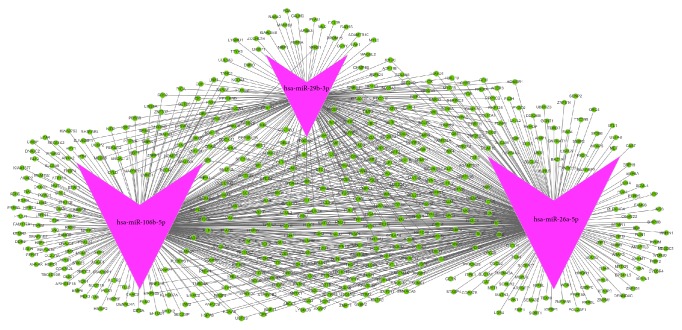
Prediction of mRNA targets regulated by the 3 miRNA signatures. The network includes two types of nodes, the miRNAs (purple arrow) and their predicted mRNA targets (green circle), based on miRTarBase, MicroCosm, and TargetScan databases. We selected the top 10 term targets to create the mRNA-miRNA interaction network and then visualized the data using Cytoscape.

To extend our knowledge of the regulatory information network (RIN) that is associated with hsa-miR-106b, hsa-miR-26a, and hsa-miR-29b, we utilized the existing publicly available database GeneSpring 12.5 to predict the potential targets. Validated target genes were automatically obtained from the microRNA.org, PITA, and TargetScan repositories. We then selected the predicted common expression target genes from three databases and chose the top 10 term targets to create the mRNA-miRNA interaction network and then visualized the data using Cytoscape (Figure 3).

**Figure 4:**
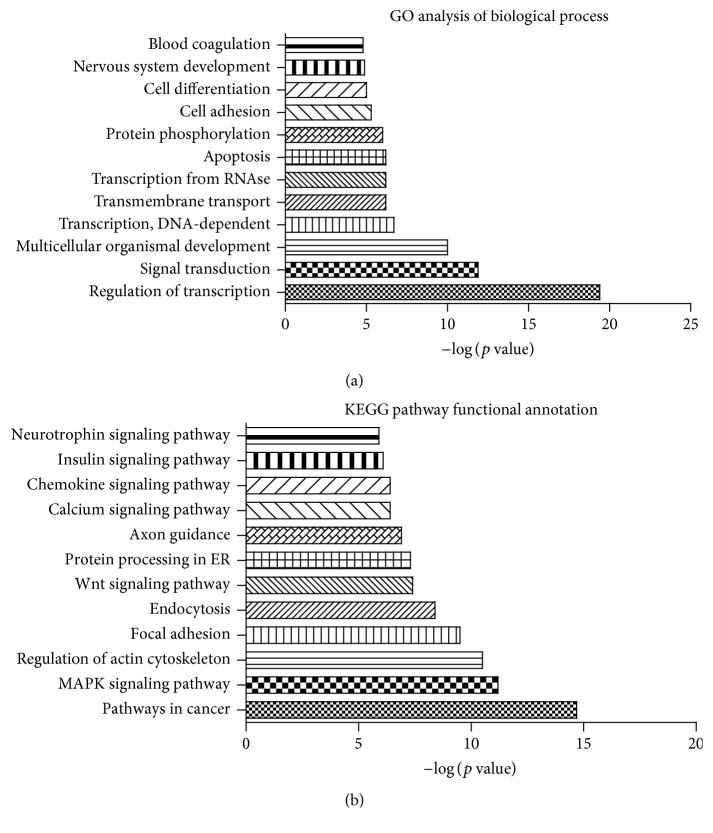
Functional annotation analysis of 3 miRNAs. GO analysis of biological process (a) and KEGG pathway functional annotation (b) of the differentially expressed miRNA (AUC values ≥ 0.90) predicted targets. Enrichment scores corresponding to each catalogue provided by the DAVID annotation tool are displayed as −log *p* values.

To obtain a better understanding of the biological functions of the three representative miRNAs in T2DM with D-IBS, GO analysis of the miRNA target genes was performed to describe the biological processes of these genes. As a result, 12 GO categories were found to be associated with T2DM with D-IBS (*p* ≤ 0.05). Among them, most of the biological processes in the whole gene set were associated with the regulation of transcription (DNA-dependent), signal transduction, multicellular organismal development, transcription (DNA-dependent), transmembrane transport, and positive regulation of transcription with the RNA polymerase II promoter (Figure 4(a)). Other GO terms of relevance were cancer adhesion and apoptosis. Taken together, the computational analysis of the validated mRNA targets for the three miRNA signatures and their associated GO terms suggested that T2DM with D-IBS is a consequence of DNA-dependent transcription and signal transduction.

In addition, the enrichment of the KEGG pathways in the target gene set was also assessed using a right-sided hypergeometric statistical analysis, which provides a *p* value for further correction using a Bonferroni step-down method. Using a *p* value cutoff of less than 0.05, pathways in cancer, MAPK signaling, Wnt signaling, calcium signaling, chemokine signaling, insulin signaling and neurotrophin signaling, regulation of actin cytoskeleton, focal adhesion, endocytosis, protein processing in the endoplasmic reticulum, and axon guidance were the significantly enriched KEGG signaling pathways and were involved in T2DM with D-IBS (Figure 4(b)).

## 4. Discussion 

We observed increased prevalence of T2DM in patients with IBS, which has seldom been reported [12]. Most studies of miRNAs in IBS focus on biopsies, which require invasive sampling of the small intestinal/colonic mucosa [21]. A similar situation also exists in diabetes [25]. Because the exact reason of T2DM with D-IBS remains unclear, the detection of novel biomarkers and their potential implications in the etiology of this disease may contribute to a better understanding of the mechanisms of the disease. In addition, it is advantageous in clinical practice, enabling more adequate management and an even earlier diagnosis, ultimately enhancing the quality of life. miRNAs are known to be implicated in a series of biological processes [26, 27], and their abnormal expression has been described in many metabolic and dysfunction diseases, including T2DM and IBS. Recently, the discovery of circulating fetal nucleic acids in maternal plasma [28] and of circulating extracellular miRNAs in the plasma of cancer patients [29, 30] has suggested a broad opportunity for the development of circulating miRNAs as blood-based markers for use in noninvasive molecular diagnostics. Therefore, plasma miRNA-based biomarkers may allow the comprehensive investigation of T2DM with D-IBS.

Few studies have assessed the miRNA expression displayed by diabetes and/or IBS patients relative to matched subjects. Zhou performed microvesicle-miRNA profiling on a subset of IBS patients with diarrhea and increased intestinal permeability which revealed upregulation of miR-29 [20]. Additionally, miR-29 is an important regulatory factor in normal metabolism and may represent a novel therapeutic target in metabolic syndromes [31, 32]. These studies also demonstrated the upregulation of hsa-miR-29b in T2DM and D-IBS. Similarly, miR-29b, a member of the miR-29 family, was found to be upregulated in the current work. Furthermore, in agreement with our results, global or liver-specific overexpression of miR-26a in mice fed high-fat diet improved insulin sensitivity and decreased hepatic glucose production and fatty acid synthesis, thereby preventing obesity-induced metabolic complications [22]. MicroRNA-106b induces mitochondrial dysfunction and insulin resistance in C2C12 myotubes by targeting mitofusin-2 [23]. However, this parameter was not decreased in the T2DM with D-IBS. The expression levels of hsa-miR-106b, hsa-miR-26a, and hsa-miR-29b were significantly elevated in T2DM with D-IBS, T2DM, and D-IBS compared to the control subjects, and the levels of hsa-miR-106b, hsa-miR-26a, and hsa-miR-29b expressions were significantly different in T2DM with D-IBS compared to either T2DM or D-IBS. In accordance with our findings, a decreased level of miR-146 expression in peripheral blood mononuclear cells is correlated with ongoing islet autoimmunity in patients with type 1 diabetes [33]. A significantly lower level of expression of miR-30 family microRNAs confers an epithelial phenotype to human pancreatic cells during the mesenchymal transition [34], while in the present study, we also observed downregulation of this miRNA in plasma from T2DM with D-IBS. None of the other differentially modulated miRNAs that were detected in our work have been reported previously in any other studies that compared T2DM with D-IBS to healthy individuals. Hence, in addition to confirming the findings of miRNAs with significantly abnormal expression in patients suffering from T2DM with D-IBS, we identified novel dysregulated miRNAs in T2DM with D-IBS relative to the control subjects. In summary, our findings identified three miRNA species, hsa-miR-106b, hsa-miR-26a, and hsa-miR-29b, which are differentially expressed in the peripheral circulation of patients suffering from T2DM with D-IBS.

The integrated results of the GO analysis of three miRNAs (hsa-miR-106b, hsa-miR-26a, and hsa-miR-29b) suggested that transcription [regulation of transcription (DNA-dependent), transcription (DNA-dependent), and positive regulation of transcription by the RNA polymerase II promoter] and signal pathways (signal transduction, transmembrane transport, and protein phosphorylation) are the most important GO terms related to the aggression of T2DM with D-IBS. The results were confirmed by GO map analysis, which can systemically construct the interaction network of the significant GO terms. In addition, in the present study, we discovered that the target genes in this RIN of KEGG were mainly involved in pathways in MAPK signaling. In some studies, a higher occurrence of functional bowel symptoms was observed in patients with diabetes compared with control groups, and autonomic dysfunction was hypothesized to explain this observation [35]. Those results suggested the pathogenesis of T2DM with D-IBS to be related to the MAPK signaling pathway. Glucose intolerance in MKP5-deficient mice is accompanied by a significant increase of visceral adipose weight, reduced AKT activation, enhanced p38 activity, and increased inflammation in visceral adipose tissue when compared with wild-type (WT) mice [36]. The significantly aberrant MAPK signaling and the differential expressive levels of the MAPK14 gene in both insulin-sensitive tissues suggested a common role of the p38-MAPK-dependent mechanism in the pathophysiology of T2DM [37]. Zhou et al. examined the expression of miR-29 and demonstrated that it was higher than that in the control group, which reduced expression of CLDN1 and NKRF to increase intestinal permeability [21]. Binion examined the effect of curcumin and MAPK inhibitors on COX-2 gene expression and angiogenesis in HIMECs following VEGF stimulation and showed that COX-2 plays a vital role in VEGF-induced angiogenesis via MAPKs and that curcumin blocks both COX-2 expression and angiogenesis which are induced by VEGF [38]. Additionally, diabetes was common in patients with D-IBS, which suggested that the MAPK pathway might be responsible for the progression to T2DM in patients with D-IBS.

In conclusion, the current study identified a set of 35 differentially expressed miRNAs that bear the potential to be molecular markers of T2DM with D-IBS, as they clearly discriminated T2DM with D-IBS from healthy subjects. Furthermore, our findings identified three miRNA species, hsa-miR-106b, hsa-miR-26a, and hsa-miR-29b, which are differentially expressed in the peripheral circulation of patients suffering from T2DM with D-IBS and are among the miRNAs that are implicated in the MAKP signaling pathway. Further research will reveal the specific roles of hsa-miR-106b, hsa-miR-26a, and hsa-miR-29b in this pathway and whether they could be developed as biomarkers and/or therapeutic targets in T2DM with D-IBS.

## Figures and Tables

**Table 1:** Characteristics of IBS and healthy subjects.

	IBS (*n* = 201)	Control (*n* = 220)	*p* value
Age	40.7 ± 6.2	39.2 ± 7.3	*p* > 0.05
Sex (% woman)	63.7	58.2	*p* > 0.05
BMI	24.6 ± 2.1	24.8 ± 2.3	*p* > 0.05
D-IBS (%)	63.7%	—	—
C-IBS (%)	36.3%	—	—
Fasting blood glucose (mmol/L)	6.84 ± 1.15	5.4 ± 1.06	*p* < 0.05

**Table 2:** Primer sequences for the real-time RT-PCR for miRNAs and U6.

Gene name	Primer	Sequence (5′ >3′)
miR-106b	Forward primer	CTCAACTGGTGTCGTGGAGTCGGCAATTCAGTTGAGATCTGCA
	Reverse primer	ACTGCTAAAGTGCTGACAGTGCA
miR-26a	Forward primer	CTCAACTGGTGTCGTGGAGTCGGCAATTCAGTTGAGAGCCTAT
	Reverse primer	ACTGCTTCAAGTAATCCAGGATA
miR-29b	Forward primer	CTCAACTGGTGTCGTGGAGTCGGCAATTCAGTTGAGAACACTG
	Reverse primer	ACTGCTAGCACCATTTGAAATCA
U6	Forward primer	CTCGCTTCGGCAGCACA
	Reverse primer	AACGCTTCACGAATTTGCGT

**Table 3:** Differentially expressed miRNAs in T2DM with D-IBS compared with D-IBS and T2DM.

Systematic name	Fold change	Regulation	*p* value
hsa-let-7d-3p	5.77	Down	0.007
hsa-miR-106b-5p	105.47	Up	1.15*E* − 04
hsa-miR-122-5p	34.07	Up	0.019
hsa-miR-1260a	8.43	Down	0.003
hsa-miR-144-3p	7.75	Down	0.048
hsa-miR-146a-5p	13.22	Down	4.79*E* − 05
hsa-miR-148b-3p	6.32	Up	0.002
hsa-miR-151a-3p	32.78	Down	6.53*E* − 04
hsa-miR-1587	125.56	Down	0.013
hsa-miR-19b-3p	191.08	Up	1.17*E* − 04
hsa-miR-2116-3p	5.50	Down	4.24*E* − 04
hsa-miR-221-3p	9.43	Down	1.73*E* − 04
hsa-miR-26a-5p	140.31	Up	5.04*E* − 05
hsa-miR-29b-3p	4.75	Up	0.003
hsa-miR-301a-3p	6.63	Down	0.003
hsa-miR-30a-5p	9.75	Down	8.94*E* − 04
hsa-miR-30d-5p	9.76	Down	0.030
hsa-miR-3135b	42.63	Down	0.006
hsa-miR-320d	82.36	Down	0.015
hsa-miR-320e	89.08	Down	0.015
hsa-miR-324-3p	63.32	Down	5.55*E* − 05
hsa-miR-338-3p	14.23	Down	0.004
hsa-miR-361-5p	4.36	Down	2.12*E* − 04
hsa-miR-3620-3p	17.72	Down	6.35*E* − 04
hsa-miR-3676-5p	9.16	Down	8.72*E* − 05
hsa-miR-409-3p	19.17	Down	0.011
hsa-miR-4286	5.81	Down	0.003
hsa-miR-4428	7.95	Down	0.002
hsa-miR-4454	6.48	Down	0.007
hsa-miR-4664-3p	309.25	Down	4.92*E* − 05
hsa-miR-4785	6.14	Down	4.12*E* − 04
hsa-miR-486-3p	10.52	Down	0.004
hsa-miR-5100	3.81	Down	0.007
hsa-miR-572	7.85	Up	1.22*E* − 04
hsa-miR-92a-3p	96.30	Down	0.048
